# RGD delivery of truncated coagulase to tumor vasculature affords local thrombotic activity to induce infarction of tumors in mice

**DOI:** 10.1038/s41598-017-05326-9

**Published:** 2017-08-15

**Authors:** Rana Jahanban-Esfahlan, Khaled Seidi, Hassan Monhemi, Amir Daei Farshchi Adli, Babak Minofar, Peyman Zare, Davoud Farajzadeh, Safar Farajnia, Ramezan Behzadi, Mehran Mesgari Abbasi, Nosratollah Zarghami, Tahereh Javaheri

**Affiliations:** 1 Department of Medical Biotechnology, Faculty of Advanced Medical Sciences, Tabriz University of Medical Sciences, Tabriz, Iran; 2 Structural Biology and Bioinformatics Research Group, Khayyam Bioeconomy Institute (KBI), Mashhad, Iran; 3 Faculty of Veterinary Sciences, The University of Melbourne, Werribee, Victoria Australia; 4 Department of Cellular and Molecular Biology, Faculty of Biological Science, Azarbaijan Shahid Madani University, Tabriz, Iran; 5 Drug Applied Research Center, Tabriz University of Medical Sciences, Tabriz, Iran; 6 North Research Center, Pasture Institute of Iran, Tehran, Amol Iran; 7 Faculty of Science, University of South Bohemia, Branišovská 1760, 37005 České, Budějovice Czech Republic; 8 Center for Nanobiology and Structural Biology, Institute of Microbiology, Academy of Sciences of the Czech Republic, Zámek 136, 373 33 Nové Hrady, Czech Republic; 9 University of Veterinary Medicine, Ludwig Boltzmann Institute for Cancer Research, Institute for Animal Breeding and Genetics, Veterinärplatz 1, A-1210, Vienna, Austria; 10 Department of Clinical Biochemistry and Laboratory Medicine, Faculty of Medicine, Tabriz University of Medical Sciences, Tabriz, Iran

## Abstract

Induction of thrombosis in tumor vasculature represents an appealing strategy for combating cancer. Herein, we combined unique intrinsic coagulation properties of staphylocoagulase with new acquired functional potentials introduced by genetic engineering, to generate a novel bi-functional fusion protein consisting of truncated coagulase (tCoa) bearing an RGD motif on its C-terminus for cancer therapy. We demonstrated that free coagulase failed to elicit any significant thrombotic activity. Conversely, RGD delivery of coagulase retained coagulase activity and afforded favorable interaction of fusion proteins with prothrombin and α_v_β_3_ endothelial cell receptors, as verified by *in silico, in vitro*, and *in vivo* experiments. Although free coagulase elicited robust coagulase activity *in vitro*, only targeted coagulase (tCoa-RGD) was capable of producing extensive thrombosis, and subsequent infarction and massive necrosis of CT26 mouse colon, 4T1 mouse mammary and SKOV3 human ovarian tumors in mice. Additionally, systemic injections of lower doses of tCoa-RGD produced striking tumor growth inhibition of CT26, 4T1 and SKOV3 solid tumors in animals. Altogether, the nontoxic nature, unique shortcut mechanism, minimal effective dose, wide therapeutic window, efficient induction of thrombosis, local effects and susceptibility of human blood to coagulase suggest tCoa-RGD fusion proteins as a novel and promising anticancer therapy for human trials.

## Introduction

Myocardial/brain infarctions are the leading causes of death around the globe. A sole blood clot is enough to cause congestion of a single vessel, and further deplete cells of oxygen and nutrients. Sustained energy depletion in infarctive tissue leads to necrosis and subsequent death of cells^1^. A similar strategy could be adopted to combat cancer by generating selective tumor vascular coaguligands, consisting of a coagulation factor plus a tumor vascular targeting moiety^2^. Since 1997, a truncated form of human tissue factor (tTF) has been linked to different tumor endothelial homing tags, including antibodies against MHC-II, vascular cell adhesion molecule-1 (VCAM-1), NG2 proteoglycan, EDB domain of fibronectin, prostate specific membrane antigen (PSMA), vascular endothelial growth factor receptor and short amino acid sequences containing Arg-Gly-Asp (RGD)/Asn-Gly-Arg (NGR) tri-peptides for targeting α_v_β_3_ integrins^2–10^. Since α_v_β_3_ integrins are abundantly and exclusively expressed by tumor endothelial cells and are absent from the vasculature of normal organs, they confer a rational target for cancer therapy^7, 11^. Accordingly, TF fusion proteins such as tTF-RGD are demonstrated to induce selective, fast and widespread thrombosis in tumor vasculature, leading to the rapid shutdown of tumor blood flow and subsequent necrosis and destruction of tumor cells^12^.

Although induction of tumor vascular infarction by tTF fusion proteins leads to the significant regression of malignant tumors, and could even bear curative potential as single therapies^13^, complete elimination of tumors often fails due to induction of incomplete thrombosis and the systemic toxicity that is reported for tTF fusion proteins at the higher doses^14^.

Among coagulation factors, staphylocoagulase (SC) from *S. aureus* represents a unique mechanism^15^. Coagulase does not cleave prothrombin (ProT) to thrombin (T)^16^. Instead, coagulase acts as a cofactor, inducing a conformational change in prothrombin to produce an active complex for converting fibrinogen to fibrin^17^. This unusual non-enzymatic activation by inducing conformational changes in zymogens is called “molecular sexuality mechanism”, which is described for the staphylocoagulase family of Zap Activated Adhesion Proteins (ZAAPs)^18^. SC binds to both prothrombin and thrombin, forming complexes that specifically cleave fibrinogen to fibrin, but not the other biological thrombin substrates, platelets, factor V or factor VIII^17^. Thus, platelet aggregation which is a characteristic feature of regular coagulation is expendable for coagulase mediated thrombosis. Notably, SC-ProT complexes are resistant to conventional anticoagulants such as heparin cofactor II, antithrombin-heparin, and plasma serpins^17^. As coagulase directly recruits the last partners of coagulation, prothrombin, and fibrinogen, it bypasses the regular coagulation pathway and exerts local yet efficient thrombosis without activating the other clotting factors^19–21^.

Herein, we combined unique intrinsic coagulation properties of staphylocoagulase with new acquired functional potentials introduced by genetic engineering, to generate a novel fusion protein consisting of truncated coagulase (tCoa) bearing an RGD motif on its C-terminus, to induce selective infarction of tumor-feeding blood vessels for cancer therapy. We demonstrated that RGD mediated localization of bacterial coagulase was indispensable for thrombogenic activity and elicited a robust and localized vascular thrombosis and infarction of different established tumors in mice. This study describes the first application of engineered bacterial coagulase as a novel and promising anticancer therapy.

## Results

### Cloning, expression, and characterization of the fusion proteins

**Figure 1:**
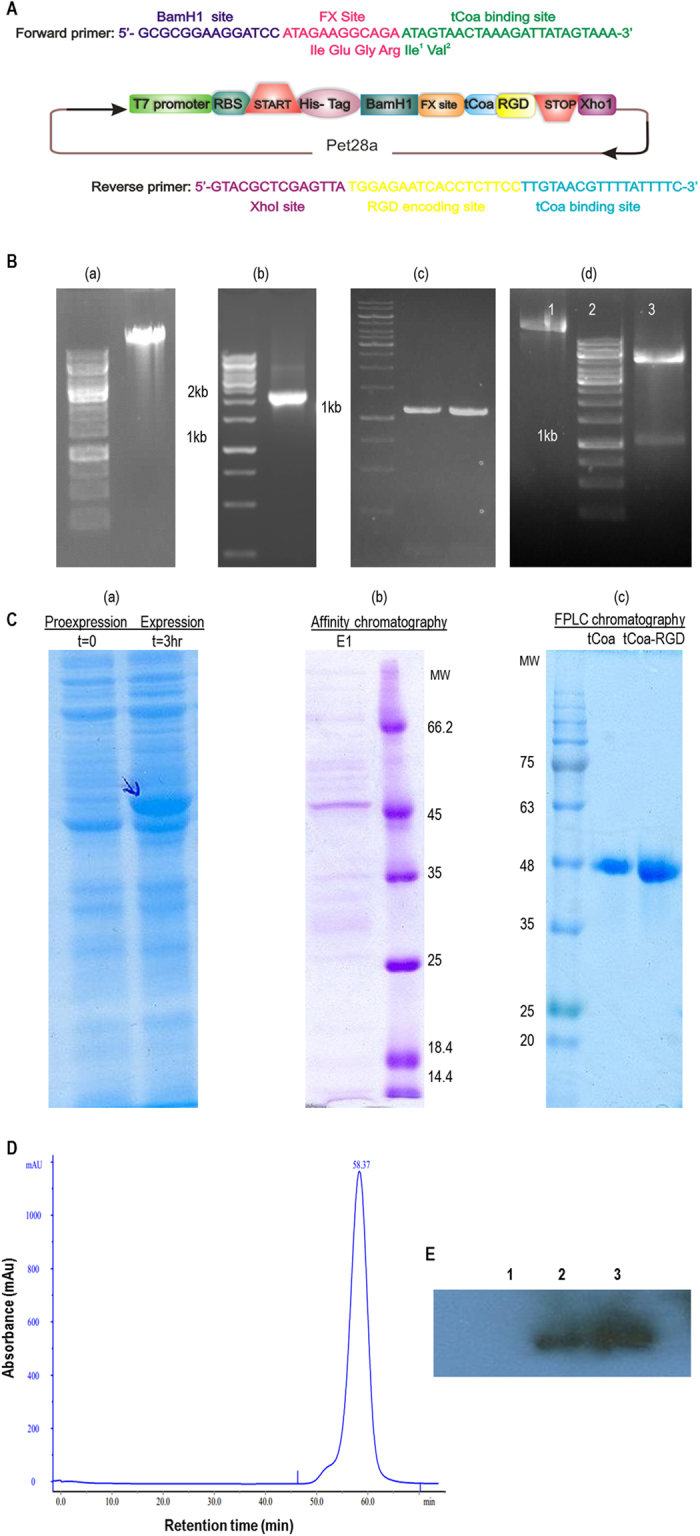
The design of the coagulase gene construct, isolation, cloning, expression, purification and identification of the corresponding fusion proteins. (**A**) The design of tCoa-RGD gene constructs for cloning. The addition of Factor X site to the 5′ end of coagulase retained the order of Ile-1-Val-2, which is critical for full coagulase activity. Factor Xa cleaves after the arginine residue in its preferred cleavage site Ile-(Glu or Asp)-Gly-Arg. (**B**) (a) genomic DNA extraction, (b) amplification of complete coagulase gene (~2 Kbp), (c) amplification of 1.2 Kbp gene constructs, and (d) cloning of tCoa-RGD into the pet 28-a vector: (1) undigested plasmid, (2) size marker, and (3) double digested (XhoI, BamH1) plasmid. (**C**) SDS-PAGE analysis of tCoa- RGD (a) pro-expression (time = 0) and expression (time = 3 h), (b) after purification with NiNTA chromatography, (c) after purification with FPLC. (**D**) FPLC analysis of purified tCoa-RGD. The peck corresponds to a ~45 kDa single protein with ~60 min retention time. (**E**) Western blotting analysis of tCoa-RGD: (1) before protein induction, (2) after protein induction, and (3) after purification, Abbreviations: T = time, E1 = elute1, MW = molecular weight.

The design of the truncated coagulase-RGD gene construct is graphically shown in Fig. 1A. First, we employed genomic DNA of a native strain of *staphylococcus aureus* (ATCC 29213) to isolate a ~2kbp fragment encompassing a complete coagulase gene (Fig. 1B). Next, based upon the sequence of 2 Kbp fragments, specific primers for PCR amplification of ~1.2 Kbp gene constructs coding for tCoa and tCoa-RGD were designed (Fig. 1B). The Ile-1-Val-2 on N-terminus of coagulase is indispensable for full coagulase activity. Upon restricted digestion, two residual nucleotides were added to the 5′ end of the ATAGTA sequence coding for Ile-1-Val-2. In order to resolve this problem, a factor X site was introduced before the sequence coding for Ile (ATA) to maintain proper interaction of coagulase with prothrombin (Fig. 1A).

We constructed and expressed a fusion protein consisting of truncated coagulase (tCoa) harboring an RGD motif of GRGDSP on its C-terminus and a poly-His-tag on its N-terminus (Fig. 1). SDS-PAGE analysis showed that protein was expressed in the supernatant phase following bacterial lysis, which indicates the solubility and cytoplasmic expression of the recombinant protein (Fig. 1C). After purification by NiNTA column and SDS-PAGE, a single band the size of about 45 kDa was detected for both tCoa and tCoa-RGD proteins (Fig. 1C). The purity of the fusion protein was also confirmed by FPLC, which showed a corresponding single peak with a retention time of ~60 min (Fig. 1D). Consequently, employing anti-6X His-tag antibody (Sigma), the identity of tCoa-RGD fusion proteins were confirmed by western blotting (Fig. 1E).

### Functional studies of recombinant tCoa-RGD proteins

**Figure 2:**
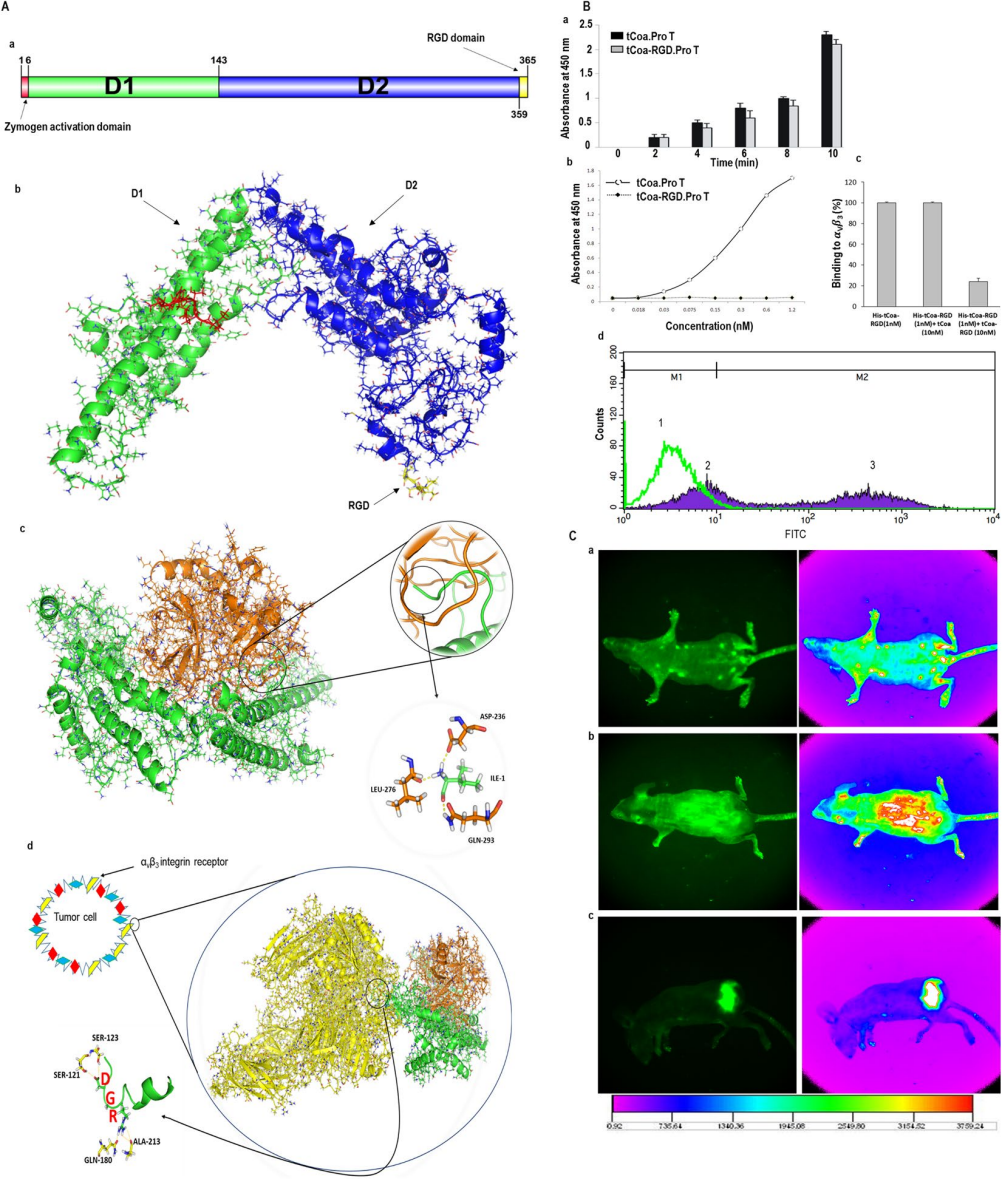
Functional studies of tCoa-RGD fusion proteins. (**A**) Modeling, docking, and MD simulation. (a) Amino acid sequence of tCoa-RGD, its domains, and 3D structure. (b) tCoa-RGD has a helical structure within its two domains. (c) The 3D structure of tCoa-RGD in complex with prothrombin after equilibration in MD simulation. Insertion of tCoa-N-terminal into prothrombin is highlighted by a circle. IVTKDY hexapeptide at the N-terminus of protein interacts strongly with zymogen activation domain of prothrombin in a “molecular sexuality mechanism.” (d) The 3D structure of tCoa-RGD-prothrombin in complex with the extracellular domain of α_v_β_3_ integrins after equilibration in MD simulation. The interaction of RGD domain with integrin residues is highlighted with a circle. (**B**) Functional studies to determine coagulase activity of the fusion proteins (a), and retention of antibody activity by tCoa-RGD to α_v_β_3_ integrins by ELISA (b, c) and FACS (d). (a) Retention of coagulase activity was determined by the capability of tCoa and tCoa-RGD to convert fibrinogen into fibrin through the conformational activation of ProT. tCoa-RGD presented coagulase activity comparable to that of tCoa at each time point, whereas ProT alone did not potentiate coagulation and there was no detectable absorbance. (b) *In vitro* binding studies demonstrated that tCoa-RGD specifically bind to immobilized α_v_β_3_ integrins in a concentration dependent manner, with the highest binding at 1.2 nM concentration. (c) In presence of His-tag removed tCoa-RGD but not tCoa, binding of tCoa-RGD to the α_v_β_3_ integrins was significantly inhibited (>70%). (d) FACS analysis verified differential binding of tCoa (peck 1) and tCoa-RGD (peck 2, 3) on endothelial cells in suspension. In this histogram, M1 marker covers the negative cells presenting no/none-specific binding while M2 marker comprises the positive cells. Accordingly, specific binding of tCoa-RGD to the α_v_β_3_ integrins was determined 64.24% (peck 3). None-specific binding for tCoa and tCoa-RGD was measured 98% and 36.05%, respectively. Correspondingly, the measured fluorescence intensity for tCoa-RGD was eight times higher than that of tCoa. (**C**) Tracing of fluorescently labeled drugs *in vivo*. tCoa-RGD labeled with FITC injected to (a) healthy mice with no tumor; while (b) tCoa and (c) tCoa-RGD injected to mice bearing SKOV3 ovarian carcinoma xenografts. tCoa-RGD fusion protein but not tCoa showed specific accumulation at the subcutaneously implanted tumor site in C57Bl/6 nude mice.

As shown in Fig. 2, functional studies to determine enzyme activity and selective binding to prothrombin and α_v_β_3_ integrin receptors were performed *in silico, in vitro, and in vivo*.

The structure of our recombinant protein was characterized by molecular modeling. Amino acid sequence and 3D structure of tCoa-RGD which were obtained after modeling and MD simulation equilibration are graphically presented in Fig. 2. As shown in the Figure, tCoa-RGD predominately consists of a helical structure which is in accordance with the other coagulase variant crystal structures^15^. The structure can be divided into two helical domains D1 and D2 (Fig. 2A(a)). D1 and D2 are separated by an angle of about 105° which is similar to that obtained in the experimental work of Friedrich *et al*.^15^ Helixes of coagulase are important structural features for its binding to targets and its zymogen activation mechanism. The helical contents of our truncated coagulase remain robust and stable during MD simulation, which shows the overall stability of protein conformation. As shown in Fig. 2A(b), RGD at C-terminus of tCoa is freely available to bind to its target which guarantees the binding to α_v_β_3_ integrins on tumor endothelial cells.

To elucidate the molecular mechanism of interaction between tCoa and prothrombin, and also to construct a predictive model for the possible favorable interactions, we applied an extensive molecular docking and MD simulation for these interacting macromolecules. The 3D structure of tCoa-RGD-prothrombin complex after docking and 20 ns equilibration is graphically represented in Fig. 2A(c). As shown in this Figure, two proteins form a tight and well-structured complex. The crystal structure of another staphylocoagulase variant (Newman) in complex with prothrombin has been experimentally solved by Friedrich *et al*.^15^ Surprisingly, the tCoa-RGD-prothrombin, which derived from our MD simulation, resembles most of the structural features in their experimental results. The most important characteristic of the tCoa-RGD-prothrombin complex is the role of N-terminal in zymogen activation. As shown in Fig. 2A(c), the N-terminal of tCoa-RGD inserts into the prothrombin activation cavity through its first hexapeptide Ile-1 to Tyr-6. The α-ammonium group of Ile-1 interacts strongly with negatively charged groups of Asp-236 and its neighboring carbonyls in prothrombin. This finding is in agreement with “zymogen activation through molecular sexuality” hypothesis^22^.

The binding of the tCoa-RGD-prothrombin complex with targeted surface receptors of tumor endothelial cells was studied with docking and simulation. The complex structure of the extracellular domain of α_v_β_3_ integrin and tCoa-RGD-prothrombin after initial docking and consequence MD simulation is shown in Fig. 2A(d). The complex was initially constructed through molecular docking and the output structure was submitted to 20 ns MD simulation equilibration to establish more real interactions. The important feature of the complex is the hydrogen bond interactions of SER-121 and SER-123 in integrin chain A with ASP (D) residue and also GLN-180 and ALA-213 in integrin chain B with ARG (R) residue of RGD domain of tCoa. This result is in agreement with the experimental result of Xiong *et al*.^23^ These features of our truncated coagulase, along with its structural stability, prompted us to test this thrombogen experimentally.

In order to verify coagulase activity of the fusion protein, mouse blood was treated with tCoa-RGD, tCoa or PBS. Citrated blood supplemented with PBS did not coagulate for infinite time, whereas addition of tCoa or tCoa-RGD fusion proteins effectively enhanced plasma coagulation within 45–60 minutes, indicating that the addition of RGD sequence to the C-terminal of tCoa did not affect the coagulation activity of the fusion protein.

It is reported that coagulase is capable of activating prothrombin at very low concentrations as small as 1 × 10^−16^ M when an equimolar concentration is mixed with prothrombin^21^. Our results showed that tCoa-RGD was able to activate prothrombin at 1:1 nanomolar concentrations, comparable to that of tCoa activation abilities at each time point. In contrast, no absorbance was recorded for the controls that were solely treated with prothrombin at any given time points (Fig. 2B(a)).

The specific binding of tCoa-RGD with α_v_β_3_ integrin receptors was measured by ELISA and FACS. Referring to the experiments reported by Bieker *et al*.^3^, we employed HUVEC cell line expressing high levels of α_v_β_3_ integrins and His-tag free fusion proteins as inhibitory ligands for binding studies with ELISA. As shown in Fig. 2B(b), no absorbance was recorded for tCoa, lacking anti-α_v_β_3_ motif. In contrast, at 1.2 nM concentration, tCoa-RGD presented the highest binding activity with α_v_β_3_ integrins in a dose-dependent manner. Accordingly, differential binding and specific interaction of RGD motif was ascertained by competition with the RGD motif (tCoa-RGD fusion proteins without His-tag). In presence of 10 molar fold excess of the tCoa-RGD, binding of his-tCoa-RGD was diminished below 30%, whereas 10 molar fold excess of tCoa had no inhibitory effect on His-tCoa-RGD binding, showing the high specificity and selectivity of RGD modified coagulase to the endothelial cells compared to the free protein (tCoa) (Fig. 2B(c)).

Additionally, fluorescence-activated cell sorting analysis revealed differential binding of tCoa and tCoa-RGD on microvascular cells in suspension (Fig. 2B(d)). Correspondingly, tCoa-RGD presented ~65% specific binding to the α_v_β_3_ integrins. Likewise, the fluorescence intensity for tCoa-RGD was measured to eight times higher than that of tCoa.

FITC-labeled proteins were applied to trace biodistribution of fusion proteins in the body of C57Bl/6 nude mice bearing SKOV3 tumor xenografts. One hour after injection of drugs, FITC- conjugated tCoa-RGD were selectively and exclusively accumulated in the site of SKOV3 tumor transplants in mice, whereas no significant fluorescent intensity was detected in other parts of the mice. Accordingly, mice that were injected with tCoa, or saline, showed no significant fluorescent enrichment in the tumor site or other organs (Fig. 2C).

### Therapeutic efficacy of tCoa-RGD fusion proteins

The therapeutic performance of tCoa-RGD fusion proteins was evaluated in Balb/c mice bearing 4T1 mammary cancer, CT26 colon carcinoma, and C57BL/6 nude mice bearing human SKOV3 ovarian cancer xenografts, for reproducibility.

**Figure 3:**
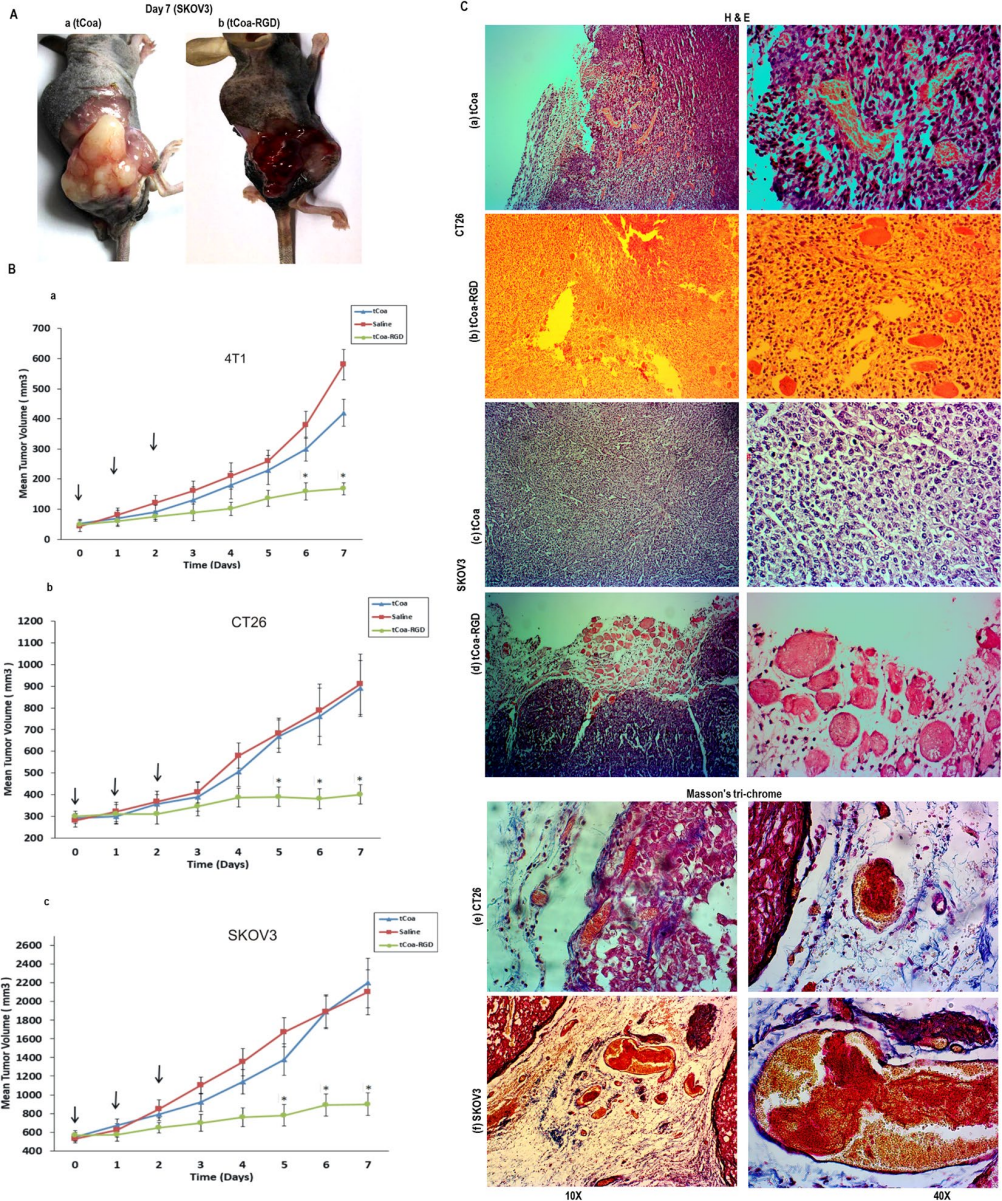
Efficacy of tCoa-RGD fusion proteins in mice. (**A**) Representative photographs of mice bearing malignant ovarian cancer (SKOV3) xenografts treated with tCoa (a) or tCoa-RGD (b) at the end of treatment. (**B**) Tumor growth inhibition studies to demonstrate therapeutic efficacy of tCoa-RGD fusion proteins. (a) 4T1, (b) CT26 and (c) SKOV3 tumor-bearing mice were injected i.v with one of the following: saline, 15 µg tCoa, 15 µg tCoa-RGD. The treatment was repeated 24 hours and 48 hours after the first injection (the arrows). In all three type of solid tumors, a significant tumor growth inhibition was demonstrated for a group of animals that were injected with three consecutive doses of tCoa-RGD, compared to tCoa and saline groups (*P < 0.05). (**C**), Histological staining of CT26 colon carcinoma and SKOV3 human ovarian carcinoma tumors treated with tCoa and tCoa-RGD coaguligand. Figures a–e show H&E staining of (a,b) CT26 and (c,d) SKOV3, as well as Masson’s trichrome staining of (e) CT26 and (f) SKOV3 tumor sections. (a,c) Histological tumor sections of mice treated with tCoa showed no sign of thrombosis, blood vessels were either intact, containing red blood cells (a), or invisible (c), meanwhile tumor cells appeared vital. (b) Injection of tCoa-RGD resulted in thrombosis of the blood vessels distinguishing with blurred outlines, which was accompanied by disintegration and necrosis of tumoral cells (the white arrows). An example of thrombosed vessel is shown with an arrow and the thrombotic area is highlighted with a circle. (d) Likewise, in SKOV3 tumor xenografts, tCoa-RGD resulted in massive occlusion of the tumor blood vessels, including the rim area. Induction of thrombosis was highlighted by the formation of apparent mesh networks of fibrin (an example of fibrin mesh network is shown with an arrow). Induction of complete thrombosis was affirmed by deposition of fibrin and RBCs in (e) CT26 and (f) SKOV3 tumor histological sections. Red staining represents fibrins while yellow staining indicates the RBCs.

Upon administration of tCoa-RGD fusion proteins, macroscopic signs of thrombosis were detected at the skin site of tumors in mice bearing SKOV3 xenografts (Fig. 3B), indicating that tCoa-RGD did mediate a selective blood coagulation in tumor blood vessels, as previously reported by TF fusion proteins^1–9^.

Moreover, in both studied animal settings that were treated with three consecutive doses of tCoa-RGD fusion proteins, a significant decrease in tumor size was detected. Administration of tCoa-RGD fusion proteins produced a statistically significant reduction in tumor growth rate in mice bearing different solid tumors (Fig. 3B). Conversely, in control animals that were treated with three 15 µg doses of tCoa at 24-hour intervals or normal saline (200 µl), tumors tended to grow progressively.

Induction of selective thrombosis was also confirmed by histological analysis. After injection of tCoa, tumor cells were viable, and blood vessels were healthy, filled with RBCs (Fig. 3C.a) or invisible (Fig. 3C.c). In contrast, the histological sections of animals that were treated with tCoa-RGD fusion proteins displayed widespread thrombosis of tumor blood vessels in both CT26 and SKOV3 tumor models. Induction of thrombosis was highlighted with packed erythrocytes, a blurred outline, and formation of mesh networks of fibrin (Fig. 3C(b) and Fig. 3C(d)). Additionally, the disintegration of tumor cells and signs of necrosis were apparent around the thrombotic area (Fig. 3C(b)). Furthermore, Masson’s trichrome staining was employed to confirm induction of complete thrombosis in the neovasculature of CT26 (Fig. 3C(e)) and SKOV3 (Fig. 3C(f)) tumors by distinct staining of fibrin (red color) and RBCs (yellow).

**Figure 4:**
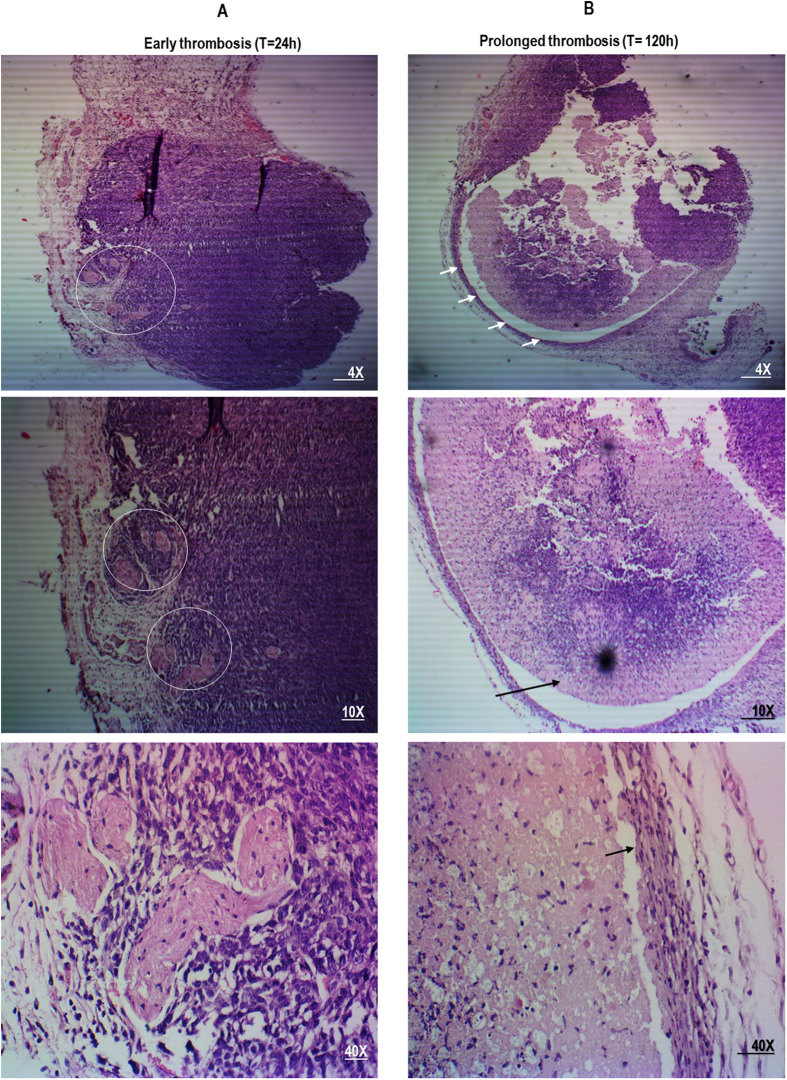
The effect of sustained induction of thrombosis by tCoa-RGD fusion proteins on 4T1 mouse mammary carcinoma tumors. Histological changes are depicted on day one and day five after the first injection. For more clarification, the tumor cross-sections are shown with three different magnifications. (**A**) Injection of tCoa fusion proteins into animal bearing mouse mammary solid tumors resulted in induction of thrombosis. Thrombotic vessels with packed erythrocytes and deposition of fibrin were apparent in stained tumor sections following injection of tCoa-RGD within 24 hours. Examples of thrombosed vessels are shown with a circle. (**B**) Induction of long-termed thrombosis by injection of three consecutive doses of tCoa-RGD at 24-hour intervals produced massive necrosis and destruction of tumor cells within the whole tumor region. Note the separation of tumor cells from each other and also complete detachment from the tumor basal membrane (the arrow).

In order to gain further insight into the therapeutic effects of long-term thrombosis on solid tumor tissues, H&E stained cross-sections of 4T1 tumors were employed. As depicted in Fig. 4, in tumor tissues that were treated with tCoa-RGD fusion proteins, the early signs of thrombosis were apparent, especially at the tumor periphery within 24 hours after a single injection, meanwhile, tumor cells surrounding the occluded vessels appeared normal (Fig. 4A). Injection of three consecutive doses of 15 µg of tCoa-RGD and sustained thrombosis resulted in disintegration and complete destruction of 4T1 tumors by day five after the first injection (Fig. 4B). As shown in this figure, tumor cells were completely detached from the basal membrane, undergoing cytolysis and necrosis, indicating that chronic dosing is required for induction of complete thrombosis and subsequent infarction in whole tumor region.

**Figure 5:**
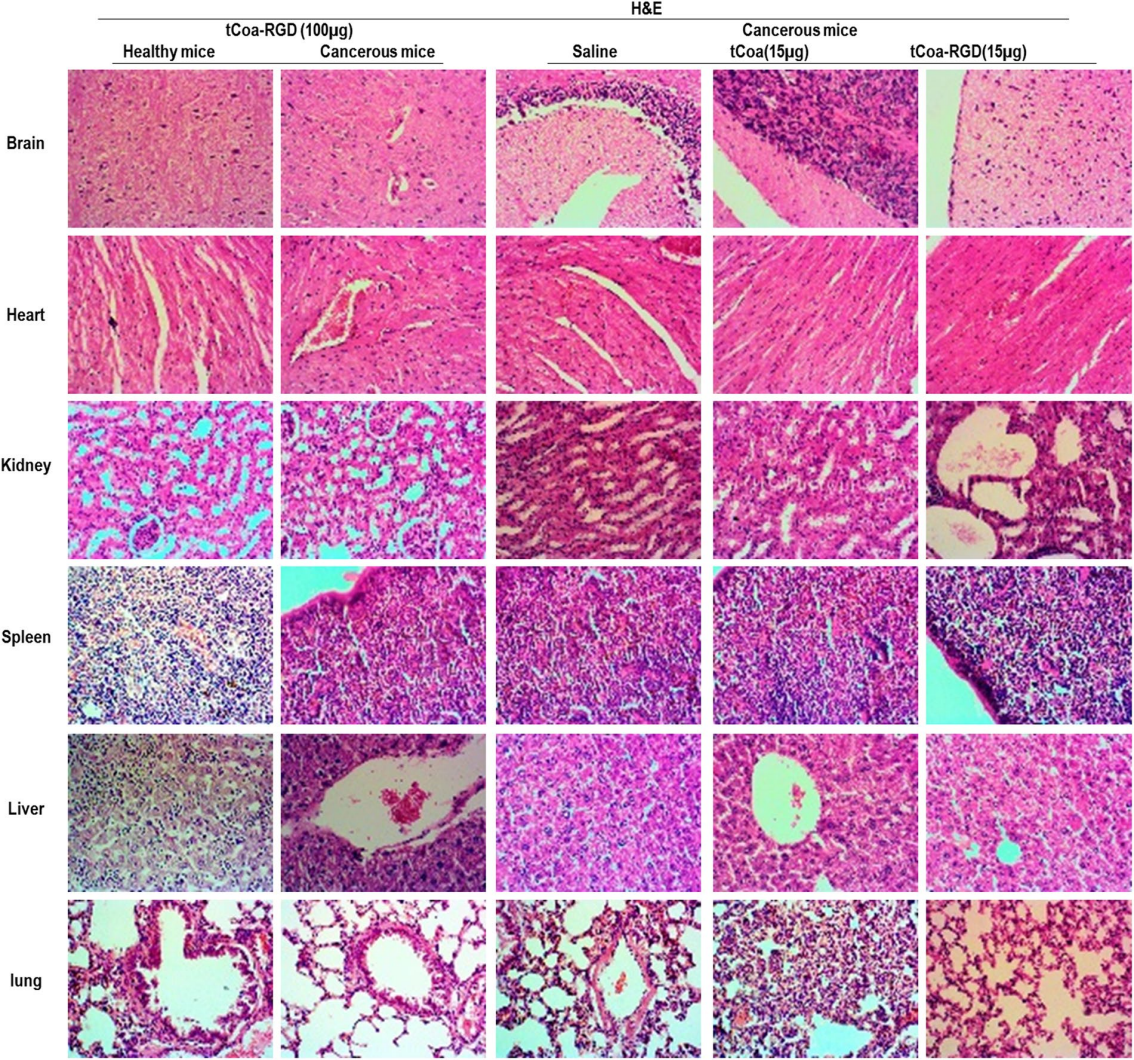
Toxicological studies in mice. This figure shows the H&E staining of organs from the healthy mice and cancerous mice treated with high dose of tCoa-RGD (100 µg). H&E staining of organs from the mice bearing human ovarian tumors treated with saline (200 µl), tCoa (15 µg), and tCoa-RGD (15 µg) is also represented. Injection of therapeutic doses, as well as high doses of the tCoa-RGD fusion protein, resulted in no sign of necrosis or thrombosis in histological analysis of vital organs including brain, heart, kidney, liver, spleen and lung, indicating the high selectivity of tCoa-RGD fusion proteins. Magnification (40x).

The drug tolerability/toxicity in mice was monitored by macroscopic (such as tail necrosis, abnormal bleeding, sudden death, weight loss) or microscopic (H&E staining) examination. Histological analysis of organs from mice bearing SKOV3 xenografts showed that compared to the control group (saline group), injection of either tCoa (15 µg) or tCoa-RGD (15 µg) resulted in no obvious organ damage or thrombosis. Accordingly, the histological analysis of normal organs obtained from cancerous and tumor-free mice that were injected with a higher dose of tCoa-RGD (100 µg), reveled no signs of thrombosis (Fig. 5). Moreover, there was no significant difference between the survival rate of healthy mice compared to the cancerous mice, indicating the high selectivity and safety of tCoa-RGD fusion proteins in mice.

## Discussion

**Figure 6:**
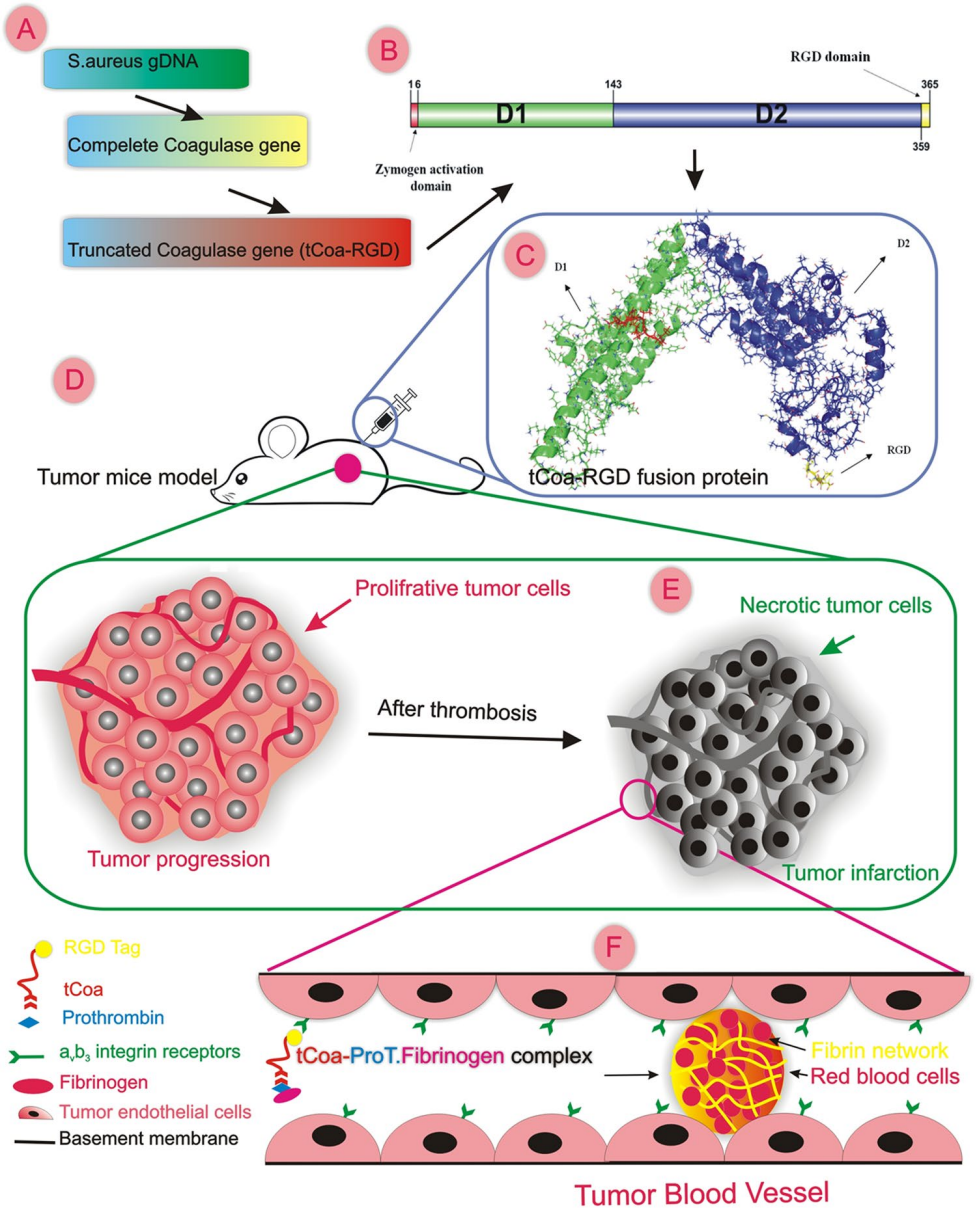
tCoa-RGD fusion proteins induce infarction of tumors in mice. This figure illustrates the main steps including design (**A**), molecular modeling (**B**), production (**C**) and the pre-clinical evaluation of the novel fusion proteins (**D**). It also highlights the unique mechanism of coagulase (**E,F**). RGD directed targeting of truncated coagulase to tumor neovasculature produces significant thrombosis and subsequent infarction and destruction of the tumor cells (**E**). Coagulase mediated coagulation is safe and efficient; this process requires the participation of only two partners, prothrombin, and fibrinogen. RGD directed delivery of coagulase to α_v_β_3_ integrin receptors on tumor endothelial cells affords the appropriate spatial localization of bacterial proteins to induce an efficient coagulation within minutes. This figure is drawn by one of our authors, it’s original and there is no need for permission.

Tumor vasculature serves as the main gateway for delivery of oxygen and nutrients to the proliferating tumor cells, thus compromising the tumor vascular system would deplete cancer cells of energy and starve them to death^24^. One appealing strategy is the induction of selective thrombosis in the pre-established tumor-feeding blood vessels by coaguligands, consisting of a coagulation factor plus an endothelial homing motif^14^. In this study, a bi-functional fusion protein was successfully constructed and employed for selective induction of thrombosis and tumor infarction in mice bearing different tumors, as a novel promising anti-cancer therapy (Fig. 6).

Panizzi *et al*. exploited a truncated form of coagulase consisting of 325aa (SC-1-325) from *staphylococcus aureus* (Newman) to study and reveal its unique crystal structure and mechanism^15^. According to Panizzi *et al*. the intact sequence of Ile-1-Val-2 on N-terminus of coagulase protein is essential for full coagulase activity, although the C-terminal tandem repeats provide the site for binding of fibrinogen, Sc 1-325 lacking in this site still possess full coagulase activity. Hence, C-terminus is suitable for molecular manipulation and tag linkage^18^. We used a truncated form of coagulase (~400aa) that was isolated from a native *staphylococcus aureus* (ATCC 29213) and linked a 6aa sequence encoding for RGD as an endothelial targeting moiety on its C-terminal. tCoa-RGD retained its favorable conformational structure to bind to prothrombin and also to α_v_β_3_ integrin receptors via its RGD tag, as confirmed by molecular dynamic analysis, as well as *in vitro* and *in vivo* experiments. Successful targeting of tTF by RGD motif of GRGDSP was previously described by Kessler *et al*.^7^ The same RGD motif was used as a suitable targeting moiety for selective directing of tCoa to α_v_β_3_ integrins by our group.

In this study, we verified that coagulase-RGD fusion proteins retained coagulase activity *in silico and in vitro*, and when targeted to the neovasculature of 4T1, CT26 and SKOV3 tumors *in vivo*, it mediated selective thrombosis of blood vessels in both medium and highly proliferative tumors, which was accompanied by massive necrosis of cancer cells. Repeated lower doses of tCoa-RGD, 24 hours and 48 hours after the first injection yielded a marked reduction of tumor mass in three different tumor models in mice. Furthermore, experiments with higher doses of coagulase (100 µg) showed no significant macroscopic or microscopic abnormalities. Additionally, there was no sign of thrombosis or necrosis in the histological sections of healthy organs of mice, indicating to the local and selective effects of our fusion protein.

Unfortunately, tumor models of mice induced by implantation of host-derived cancer cells (CT26 and 4T1 mouse cancer cells in Balb/C mice) or foreign tumor transplants (SKOV3 human xenografts in C57BL/6 nude mice) fail to present all aspects of spontaneous human tumors, including tumor biology, microenvironment, blood supply, interaction with stroma, as well as tumor growth rate and metastatic potential. Moreover, anatomic site of skin implanted tumors may provide a different microenvironment than those in naturally occurring tumors. For example, ovarian tumors located in ovaries and/or on peritoneum represent different characteristics than those tumors implanted in the subcutaneous tissue. However, regardless of the tumor microenvironment, the three type of established solid tumors in mice presented a high growth rate, a good degree of tumor vascularization, and abundant α_v_β_3_ endothelial receptors expression, thereby rendering appropriate tumor models for tCoa-RGD therapy.

It is demonstrated that equimolar concentration of coagulase suffices for activation of prothrombin. Conversely, TF fusion proteins require high concentration, for instance (RGD)_3_-tTF generated by Huanget *et al*., activated FX at 1 *μ*mol/L or higher concentration^25^. Nevertheless, tCoa fusion proteins required nanomolar concentrations of coagulase to initiate coagulation by activation of prothrombin^21^. Similarly, we obtained quite impressive results with 15 µg concentration of coagulase fusion proteins. In contrast, a 10 µg dose of TF fusion proteins is ineffective and induction of thrombosis by TF fusion proteins requires doses ≥20 µg^4^.

The thrombotic activity of impure coagulase has been evaluated in rats, humans, and rabbits. It is shown that systemically administered doses of 0.25, 0.05 and 0.12 mg of coagulase are well tolerated in rats^26^. It is noteworthy that *in vivo* administration of coagulase leaves the established blood clotting factors untouched, but with proper doses may changes fibrinogen levels. Extensive studies in Poland and in France revealed that coagulase failed to confer any considerable effect on the serum level of platelets, factors II, V, VIII, IX, X, XI, XII^27, 28^. This finding is of paramount significance, since elevated levels of these clotting factors along with the overexpression of tissue factor, plays a crucial role in balancing angiogenesis-coagulation events requisite for tumor growth, survival and metastasis^14, 29–32^. Coagulase renders an exceptional mechanism of coagulation owing to “molecular sexuality mechanism.” Coagulase bypasses coagulation cascade through conformational activation of prothrombin and avoids activation of a chain of clotting factors, accordingly coagulase mediated coagulation renders local effects.

A serious concern about the application of coagulase is its probable antigenicity in humans. An indication of the extent of this issue was given in a preliminary study conducted by Mojovic *et al*. The authors injected partially purified coagulase 39 times in 27 patients, with doses ranging from 0.013 mg to 3.9 mg per patient, by the subcutaneous, intramuscular, and intravenous routes. No reactions were encountered with 23 intravenous inoculations and neither with the other routes and doses^26^, and there was no improvement in bleeding manifestations among the patients. Defibrination might be the main concern regarding the safety of coagulase, however, this issue could be avoided by administering the appropriate doses^33^.

Our results indicated that systemically administered purified tCoa-RGD caused no treatment-related toxicity in living mice during or after cessation of the treatment. Especially, no clinical signs indicating major embolism or stroke occurred. Furthermore, lower doses of targeted coagulase resulted in impressive thrombogenic and antitumor activity. In contrast to the poor results regarding thrombotic activity of impure coagulase as a hemostatic agent in the treatment of human thrombotic disorders^26^, pure coagulase used in our experiments showed an appreciable thrombotic activity both *in vitro* and *in vivo*. It seems that targeted delivery of coagulase to the tumor vasculature restores the thrombogenic activity *in vivo*. In concordance to this notion, using microfluidics and surface patterning, Kastrup *et al*. demonstrated that the spatial localization of bacteria substantially affects coagulation of human and mouse blood, and plasma through a phenomenon called “bacteria sensing.” It was shown that *Bacillus cereus* and *Bacillus anthracis* directly initiated coagulation of blood within minutes when bacterial cells were clustered. In this regard, the quorum acting of bacteria is essential to coagulation activity. RGD delivery of bacterial coagulase to the α_v_β_3_ receptors abundantly and exclusively found in highly proliferative solid tumors affords proper localization of bacterial coagulation factor to promote rapid and local thrombosis, while free coagulase bears no significant thrombotic potential, either as a hemostatic agent or an anticancer drug. Modification of targeted coagulase by genetic engineering allowed induction of a local and robust thrombosis. This acquired feature was only attributed to RGD modified coagulase, which granted non-toxic effective doses for tCoa-RGD fusion proteins to promote coagulation. Moreover, local actions of targeted coagulase permitted a broad therapeutic window of fusion proteins. Notably, in light of the fact that human blood is more susceptible to coagulase than mice, coagulase fusion proteins would make more favorable candidates for human clinical trials.

Taken together, the nontoxic nature, unique shortcut mechanism, besides the local effects, minimal effective dose, and wide range therapeutic doses of targeted bacterial coagulase suggest tCoa-RGD fusion proteins as a novel and promising anticancer therapy.

## Materials and Methods

### Bacterial strains, cell lines, and reagents

*Staphylococcus aureus* (ATCC 29213) and tumor cell lines, including CT26 mouse colon carcinoma, 4T1 mouse mammary carcinoma cells, SKOV3 human ovarian carcinoma (SKOV3), and human umbilical vein endothelial cells (HUVECs) were purchased from the Pasteur Institute of Iran, Tehran. C57BL/6 nude mice were purchased from the Pasteur Institute of Iran, Amol. Pet28a expression vector was purchased from the Iranian Biological Resource Center (IBRC), Tehran.

### Design, production, and characterization of tCoa and tCoa-RGD fusion proteins

First, a set of primers (F1, R1) was designed to isolate a ~2Kbp fragment coding for a full-length coagulase gene from *Staphylococcus aureus* (ATCC 29213). These primers were general and could be exploited for isolation of all Genebank registered coagulase sequences. The sequence of the new 2Kb fragment was registered in GeneBank (accession number: KX914667.1) and was used to design a second set of primers for construction of a truncated form of coagulase gene (tCoa) and tCoa fused to an RGD sequence (tCoa-RGD). The sequence for the BamH1 restriction site and an additional site for factor X (FX) was placed in the F2 primer as follows:

5′-**GCGCGGAAGGATCC** ATAGAAGGCAGA *ATAGTAACTAAAGATTATAGTAAA-3*′. The bold area represents the BamHI site (GGATCC) provided with additional nucleotides to facilitate efficient enzymatic digestion. The underlined area is the FX site, which was provided to remove residual nucleotides from the N-terminus of tCoa. The italic area shows the binding site to the coagulase gene. Different reverse primers were implemented for the construction of tCoa and tCoa-RGD as follows: tCoa reverse primer: 5′-**GACTGTCACTCGAG** *CTATTAGATTTCACCTTGTAACGT-3*′. tCoa-RGD reverse primer: 5′-**GTACGCTCGAGTTA** TGGAGAATCACCTCTTCC *TTGTAACGTTTTATTTTC-*3′. The underlined area represents the sequence for RGD. XhoI restriction site (CTCGAG) was placed in the reverse primer (the bold area). The italic area represents the gene-specific binding site.

Following, the 1.2 Kbp length sequence coding for tCoa and tCoa-RGD was PCR amplified. Then, PCR products were digested with XhoI and BamHI restriction enzymes and were cloned into the pet28a vector, resulting in an expression vector consisting of (i) the RGD motif for targeting tumor blood vessels; (ii) the tCoa sequence to afford thrombogenic activation; and (iii) a 6X His-tag for further protein purification. Afterward, the tCoa-RGD fusion protein was expressed in *E.coli* strain BL21 (DE3) and purified by Ni-NTA affinity chromatography based on the manufacturer’s instruction (Qiagen). Subsequently, tCoa and tCoa-RGD were analyzed by SDS-PAGE and western blotting. Fusion proteins were subjected to Fast Protein Liquid Chromatography (FPLC) on a Superdex S-75 column (Amersham Pharmacia Biotech) to remove remaining impurities. The purity of fusion proteins was further analyzed by SDS-PAGE and FPLC.

### Functional studies of tCoa and tCoa-RGD fusion proteins

#### Molecular dynamic (MD) studies: molecular modeling, docking, and MD simulation

The structure of tCoa-RGD was modeled by I-TASSER homology modeling server^34^. The modeled structure is docked to prothrombin (PDB: 1nu9 (15)) by HADDOCK 2.2^35^. To validate the modeled structure and construction of some unstructured sequences, and also to evaluate the intramolecular interactions in dynamics mode, the complex structure was subjected to extensive MD simulation. For interaction of tCoa-RGD-prothrombin with α_v_β_3_ integrin (PDB: 1l5 g^23^), MD calculations were performed by GROMACS 5.0.1^36^ and GROMOS 54a7^37^ force field. To increase the accuracy, the simulation was repeated four times with different initial conditions. For water, the well-tested SPC/E^38^ was used. The steepest-descent algorithm was used for energy minimization of the system and relaxation of solvent molecules. LINCS^39^ and SETTLE^40^ algorithms were applied to fix the chemical bonds between the atoms of the protein and solvent molecules, respectively. The Berendsen coupling algorithm was used to retain a constant pressure and temperature for the system during simulations^41^. To estimate the electrostatic interactions, the particle mesh Ewald (PME)^42^ algorithm was applied for system components. Molecular graphics and all graphical representations were constructed by PyMOL^43^.

#### Clotting Test

Referring to coagulation experiments by McAdow *et al*.^44^, fresh mouse blood containing 1% sodium citrate was added to the plastic sterile test tubes. Next, 7 µg purified tCoa-RGD and tCoa was added to the tubes. Afterward, test tubes were incubated at room temperature (RT), and blood coagulation was confirmed by tipping the tubes to 45° angles. The samples with PBS served as control.

#### Coagulase activity

To authenticate the clotting capacities of the tCoa fragment of our fusion proteins, coagulase activity was assessed according to the experiment described by McAdow *et al*.^44^ To this, purified tCoa or tCoa-RGD (120 nM) was mixed with human prothrombin in 1% sodium citrate-PBS, and incubated at RT for 10 minutes. After an initial reading, fibrinogen (3 µM) (Sigma) was added to promote conversion of fibrinogen to fibrin. The change in turbidity at 450 nm was recorded every two minutes (BioTek). As controls, the enzymatic activity of human prothrombin was measured.

#### Binding studies

*In vitro*, ligand-receptor binding assays were performed to verify specific retention of RGD homing moiety of fusion proteins by α_V_β_3_ integrin receptors on HUVECs by enzyme-linked immunosorbent assay (ELISA) and fluorescence activated cell sorting (FACS) analysis.

Human umbilical vein endothelial cells (HUVEC) were cultured overnight in RPMI 1640 supplemented with 10% FBS in 96-well microplate at 37 C, 5% CO_2_. Then, the wells were washed with PBS. tCoa and tCoa-RGD in serial dilutions of 0, 0.018, 0.03, 0.075, 0.15, 0.3, 0.6 and 1.2 nM was added to the wells, blocked with 1% BSA and kept at RT for one hour. Then, HRP-conjugated mouse anti-poly His-tag antibody (Sigma) was added to the wells, followed by the addition of TMB coloration substrate. Absorbance at 405 nm was measured with an ELISA microplate reader (BioTek). Besides, binding steps were performed in presence of His-tag removed tCoa-RGD fusion protein as a competitive ligand to demonstrate the specificity of RGD interaction with α_V_β_3_ receptors on HUVECs.

According to our previous works^45, 46^, the specific binding of tCoa-RGD to α_V_β_3_ integrin receptors on the surface of endothelial cells was also evaluated by FACS, employing FITC conjugated anti-6X His-tag antibody (Abcam). First, microvascular endothelial cells with 80% confluency were harvested and washed twice with ice-cold FACS buffer (PBS supplemented with 10% FBS) by centrifuge at 1500 RPM. Then, cells were incubated with tCoa-RGD fusion proteins (0.15 ng/10^6^ cells) in ice cold FACS buffer for one hour at RT in the dark. After three wash steps with FACS buffer, anti-6X His tag antibody conjugated with FITC (Abcam) was added to the cells, and the reaction was incubated for 30 minutes at RT. After that, the reaction was washed twice and subjected to fluorescence-activated cell sorting analysis (BD FACS Calibur).

#### Biodistribution studies

Fluorescein isothiocyanate (FITC) conjugated proteins were prepared according to the manufacture protocol (Sigma) and were adopted for tracing of fusion proteins in the body of living mice. To this, mice bearing SKOV3 tumor xenografts were divided into two groups of tCoa and tCoa-RGD of six animals each and were injected with 35 *μ*g FITC labeled tCoa-RGD, or tCoa through the tail vein. A group of animals with no tumors (healthy group) were injected with saline, as well. One hour later, biodistribution of fluorescently labeled drugs in the body of living mice were monitored by an *in vivo* imaging system (KODAK Image Station 2000 MM).

### Therapeutic efficacy of tCoa-RGD fusion proteins in mice

#### Tumor mouse models

All animal procedures were approved and conducted in compliance with the regulations of the Laboratory Animal Ethics Committee of Tabriz University of Medical Sciences (license number: 92/4-5/4). CT26, 4T1 and SKOV3 tumor cell lines were grown to 70–80% confluency. Then, tumor-bearing mice were established by subcutaneous (s.c) injection of 2 × 10^6^ CT26 mouse colon cancer cells, 5 × 10^5^ 4T1 mouse mammary cancer cells or 5 × 10^6^ SKOV3 human ovarian carcinoma cells in 3–5 week-old female Balb/c mice or C57BL/6 nude mice, respectively. Mice were checked every day and tumor growth was calculated using a caliper according to the following formula: volume (mm^3^) = 0.52ld^2^, where l is the tumor length and d is the tumor width.

#### Treatment studies in mouse tumor models

When tumors reached certain volumes, ~50 mm^3^ for 4T1 tumors with a high growth rate and 300 mm^3^–500 mm^3^ for CT26 and SKOV3 with a medium tumor growth rate, mice were divided into three groups of saline, tCoa, and tCoa-RGD. The saline group was injected with 200 µl normal saline. In tCoa and tCoa-RGD groups, animals were intravenously given (i.v) a 15 µg concentration of purified tCoa or tCoa-RGD in 200 µl PBS on the 1st, 2nd and 3rd day of the experiment (15 µg × 3q24 hr). The animals were sacrificed on the 7th day after the first treatment.

#### Histology

To assess the extent and selective induction of thrombosis, tumor and normal organs were collected 24 hours after the injection of the tCoa-RGD fusion protein, fixed at 10% buffered neutral formalin overnight, embedded in 2% paraffin, sectioned, and stained with H&E and Masson’s trichrome as described in our previous works^24, 45–47^. Induction of thrombosis was interpreted as complete or incomplete, based on the observation of a blurred vessel outline, formation of fibrin mesh networks, the degree of closely packed erythrocytes, and fibrin deposition^6^. All experiments were carried out twice for reproducibility.

#### Toxicity studies

An extra set of animals with (cancerous mice) and without tumors (healthy mice) was injected with a higher dose of tCoa-RGD (100 µg) and monitored for 24 hours. Then, organs including brain, heart, lung, liver, spleen, and kidney were excised and analyzed by H&E staining for signs of thrombosis, hemorrhage or necrosis.

### Statistical analysis

Data presented in this study are mean ± SEM of the three independent experiments. Statistical analysis was performed by SPSS version 21. Mann-Whitney rank-sum test was employed for statistical significance difference between independent groups. *P* < 0.05 was considered significant.

### Ethics approval

The experiments were conducted strictly in compliance with the regulations of Tabriz University of Medical Sciences, Tabriz, Iran.

## References

[1] Jahanban-Esfahlan, R., de la Guardia, M., Ahmadi, D. & Yousefi, B. Modulating tumor hypoxia by nanomedicine for effective cancer therapy. *J. Cell. Physiol*, doi:10.1002/jcp.25859 (2017). 10.1002/jcp.25859 28198007

[2] Liu C (2002). Prostate-specific membrane antigen directed selective thrombotic infarction of tumors. Cancer. Res.

[3] Bieker R (2009). Infarction of tumor vessels by NGR-peptide-directed targeting of tissue factor: experimental results and first-in-man experience. Blood.

[4] Hu P (2003). Comparison of three different targeted tissue factor fusion proteins for inducing tumor vessel thrombosis. Cancer. Res.

[5] Huang FY (2008). A fusion protein containing murine vascular endothelial growth factor and tissue factor induces thrombogenesis and suppression of tumor growth in a colon carcinoma model. J. Zhejiang. Univ. Sci. B.

[6] Huang X (1997). Tumor infarction in mice by antibody-directed targeting of tissue factor to tumor vasculature. Science.

[7] Kessler T (2005). Inhibition of tumor growth by RGD peptide-directed delivery of truncated tissue factor to the tumor vasculature. Clin. Cancer. Res.

[8] Nilsson F, Kosmehl H, Zardi L, Neri D (2001). Targeted delivery of tissue factor to the ED-B domain of fibronectin, a marker of angiogenesis, mediates the infarction of solid tumors in mice. Cancer. Res.

[9] Ran S (1998). Infarction of solid hodgkin’s tumors in mice by antibody-directed targeting of tissue factor to tumor vasculature. Cancer. Res.

[10] Brand C (2016). NG2 proteoglycan as a pericyte target for anticancer therapy by tumor vessel infarction with retargeted tissue factor. Oncotarget.

[11] Liu S (2009). Radiolabeled cyclic RGD peptides as integrin alpha(v)beta(3)-targeted radiotracers: maximizing binding affinity via bivalency. Bioconjug. Chem.

[12] Camerer E, Huang W, Coughlin SR (2000). Tissue factor- and factor X-dependent activation of protease-activated receptor 2 by factor VIIa. Proc. Natl. Acad. Sci. USA.

[13] Seidi K, Jahanban-Esfahlan R, Zarghami N (2017). Tumor rim cells: from resistance to vascular targeting agents (VTAs) to complete tumor ablation. Tumor. Biol.

[14] Jahanban-Esfahlan R, Seidi K, Zarghami N (2017). Tumor vascular infarction: prospects and challenges. Int. J. Hematol.

[15] Friedrich R (2003). Staphylocoagulase is a prototype for the mechanism of cofactor-induced zymogen activation. Nature.

[16] Friedrich R (2006). Structural basis for reduced staphylocoagulase-mediated bovine prothrombin activation. J. Biol. Chem.

[17] Panizzi P, Friedrich R, Fuentes-Prior P, Bode W, Bock PE (2004). The staphylocoagulase family of zymogen activator and adhesion proteins. Cell. Mol. Life. Sci.

[18] Panizzi P (2006). Fibrinogen substrate recognition by staphylocoagulase.(pro)thrombin complexes. J. Biol. Chem.

[19] Yokota N (2014). Contributions of thrombin targets to tissue factor-dependent metastasis in hyperthrombotic mice. J. Thromb. Haemost.

[20] Nierodzik ML, Karpatkin S (2006). Thrombin induces tumor growth, metastasis, and angiogenesis: Evidence for a thrombin-regulated dormant tumor phenotype. Cancer. Cell.

[21] Cheng AG (2010). Contribution of coagulases towards Staphylococcus aureus disease and protective immunity. PLoS. Pathog.

[22] Bode W, Huber R (1976). Induction of the bovine trypsinogenâ€”trypsin transition by peptides sequentially similar to the N-terminus of trypsin. FEBS Lett.

[23] Xiong JP (2002). Crystal structure of the extracellular segment of integrin alpha V beta 3 in complex with an Arg-Gly-Asp ligand. Science.

[24] Pencik J (2015). STAT3 regulated ARF expression suppresses prostate cancer metastasis. Nat Commun.

[25] Huang ZJ (2013). Targeting the vasculature of colorectal carcinoma with a fused protein of (RGD)(3)-tTF. Sci. World. J.

[26] Mojovic B, Mojovic N, Tager M, Drummond MC (1969). Staphylocoagulase as a hemostatic agent. Yale. J. Biol. Med.

[27] Jeljaszewicz J, Niewiarowski S, Poplawski A, Prokopowicz J, Worowski K (1965). Intravascular coagulation and fibrinolysis by Staphylocoagulase. Comparision with thrombin. Thrombosis. Et. Diathesis. Haemorrhagica.

[28] Soulier JP, et Prou-Wartelle O (1967). Effets de l’injection de staphylocoagulase chez le lapin. Essais de prevention du syndrome de defibrination. Nouvelle. Revue. Franc. d’Hematologie.

[29] Ruf W, Mueller BM (2006). Thrombin generation and the pathogenesis of cancer. Semin. Thromb. Hemost.

[30] Zigler M, Kamiya T, Brantley EC, Villares GJ, Bar-Eli M (2011). PAR-1 and thrombin: the ties that bind the microenvironment to melanoma metastasis. Cancer. Res.

[31] Jiang X (2004). Formation of tissue factor-factor VIIa-factor Xa complex promotes cellular signaling and migration of human breast cancer cells. J. Thromb. Haemost.

[32] Lima LG, Monteiro RQ (2013). Activation of blood coagulation in cancer: implications for tumour progression. Biosci. Rep.

[33] Reid HA, Chan KE (1968). The paradox in therapeutic defibrination. Lancet.

[34] Yang J (2015). The I-TASSER Suite: protein structure and function prediction. Nat. Meth.

[35] van Zundert GC (2016). The HADDOCK2.2 Web Server: User-Friendly Integrative Modeling of Biomolecular Complexes. J. Mol. Biol.

[36] Lindahl E, Hess B, van der Spoel D (1995). A message-passing parallel molecular dynamics implementation. Comput. Phys. Commun.

[37] Schmid N (2011). Definition and testing of the GROMOS force-field versions 54A7 and 54B7. Eur. Biophys. J.

[38] Hermans J, Berendsen HJC, Van Gunsteren WF, Postma JPM (1984). A consistent empirical potential for water–protein interactions. Biopolymers.

[39] Hess B, Bekker H, Berendsen HJC, Fraaije JGEM (1997). LINCS: A linear constraint solver for molecular simulations. J. Comput. Chem.

[40] Miyamoto S, Kollman PA (1992). Settle: An analytical version of the SHAKE and RATTLE algorithm for rigid water models. J. Comput. Chem.

[41] Berendsen HJC, Postma JPM, van Gunsteren WF, DiNola A, Haak JR (1984). Molecular dynamics with coupling to an external bath. J. Chem. Phys.

[42] Darden T, York D, Pedersen L (1993). Particle mesh Ewald: an Nlog(N):method for Ewald sums in large systems. J. Chem. Phys.

[43] Delano, W. The PyMOL Molecular Graphics System. *Available from: citeulike-article-id:2816763*. http://www.pymol.org (2002).

[44] McAdow M (2012). Coagulases as determinants of protective immune responses against Staphylococcus aureus. Infect. Immun.

[45] Kazemi Z (2016). Repurposing Treprostinil for Enhancing Hematopoietic Progenitor Cell Transplantation. Mol. Pharmacol.

[46] Minas TZ (2015). YK-4-279 effectively antagonizes EWS-FLI1 induced leukemia in a transgenic mouse model. Oncotarget.

[47] Javaheri T (2016). Increased survival and cell cycle progression pathways are required for EWS/FLI1-induced malignant transformation. Cell death Dis.

